# Combination of PSMA targeting alpha-emitting radioligand [^212^Pb]Pb-AB001 with BET bromodomain inhibitors in in vitro prostate cancer models

**DOI:** 10.1007/s12032-025-02925-9

**Published:** 2025-07-22

**Authors:** Rugile Liukaityte, Vilde Yuli Stenberg, Andrius Kleinauskas, Petras Juzenas, Alfonso Urbanucci, Asta Juzeniene

**Affiliations:** 1 Department of Radiation Biology, Institute for Cancer Research, The Norwegian Radium Hospital, Oslo University Hospital, Oslo, Norway; 2 ARTBIO AS, Oslo, Norway; 3 Department of Physics, University of Oslo, Oslo, Norway; 4 Department of Tumour Biology, Institute for Cancer Research, The Norwegian Radium Hospital, Oslo University Hospital, Oslo, Norway; 5 Faculty of Medicine and Health Technology, TAYS Cancer Centre and FICAN Mid, Tampere University, Tampere, Finland

**Keywords:** Targeted radionuclide therapy, Targeted alpha therapy, Combination treatment, Bromodomain and extra-terminal proteins, Cell cycle

## Abstract

The alpha-emitting radioligand [^212^Pb]Pb-AB001, targeting prostate-specific membrane antigen (PSMA), is a promising therapy approach for prostate cancer. Bromodomain and extra-terminal (BET) protein inhibitors, such as AZD5153 and JQ1, disrupt oncogenic transcriptional programs by altering chromatin structure. This study evaluated whether BET inhibition enhances the efficacy of radioligand therapy. Cytotoxic effects of [^212^Pb]Pb-AB001 alone and in combination with BET inhibitors were assessed in 2D monolayers and a 3D spheroid model of PSMA-positive C4-2 prostate cancer cells. Cell viability, cell cycle alterations, and DNA damage were assessed using viability assays and flow cytometry. Spheroid growth and viability were assessed by fluorescence microscopy. AZD5153 was more potent than JQ1 in reducing cell proliferation. [^212^Pb]Pb-AB001 induced activity- and time-dependent cytotoxicity with a delayed apoptotic response. BET inhibitors induced G1 arrest, while [^212^Pb]Pb-AB001 caused G2 arrest. Combination treatment reduced cell viability in an additive manner but did not further affect cell cycle distribution or increase apoptosis compared with [^212^Pb]Pb-AB001 alone. γH2AX staining in 2D models showed an activity- and time-dependent increase in DNA damage at 1, 3 and 6 days post-treatment with [^212^Pb]Pb-AB001. BET inhibitors alone induced minimal γH2AX, and combination treatments did not enhance DNA damage beyond [^212^Pb]Pb-AB001 alone. In 3D spheroids, combination treatment led to synergistic growth suppression. In conclusion, these findings indicate that therapeutic inhibition of BET bromodomain in combination with the alpha-emitting radioligand [^212^Pb]Pb-AB001 could significantly enhance tumour control and should be further evaluated in metastatic prostate cancer models.

**Supplementary Information:**

The online version contains supplementary material available at 10.1007/s12032-025-02925-9.

## Introduction

Prostate cancer is the second leading cause of cancer-related death among men globally [1]. After an initial clinical response, prostate cancer patients often relapse and develop metastatic castration-resistant prostate cancer (mCRPC), which presents significant therapeutic challenges and remains incurable despite treatment advancements [2]. Targeted radionuclide therapy using agents targeting prostate-specific membrane antigen (PSMA), which is overexpressed in 75–90% of mCRPC cases, has emerged as a promising approach for mCRPC [3–5]. The approval of the beta-emitter [^177^Lu]Lu-PSMA-617 in 2022 has expanded its clinical use globally, offering new pathways for mCRPC management [6]. Nevertheless, only up to 60% of patients treated with [^177^Lu]Lu-PSMA-617 respond, and many initial responders eventually relapse [4, 5, 7]. Targeted alpha-particle therapies represent an attractive alternative therapeutic strategy due to their high linear energy transfer and short range, enabling effective cancer cell eradication while minimizing collateral damage to healthy tissue [8, 9]. Recent clinical studies suggest that despite prolonging the overall survival, targeted alpha and beta therapies need further clinical optimization, including combinations with other treatment modalities to enhance the efficacy and overcome resistance mechanisms [10].

Epigenetic alterations significantly influence the development and progression of mCRPC [11, 12]. Bromodomain and extra-terminal domain (BET) proteins, including BRD2 and BRD4, serve as epigenetic acetylation readers that influence tumorigenic signalling in various cancers, including prostate cancer [11, 13, 14]. Their overexpression is associated with poor clinical outcomes due to the upregulation of genes involved in growth and proliferation [12, 13]. BRD4 is involved in several oncogenic pathways such as progression through the cell cycle and DNA repair processes, specifically the non-homologous end joining pathway [15, 16]. The recruitment of BRD4 to chromatin has also been shown to increase in response to DNA damage by ionizing radiation and subsequent acetylation of histone H4 [15, 17]. It has been suggested that BRD4 then recruits other proteins required for DNA repair to the damage site [15]. Several inhibitors, such as JQ1, ZEN-3694, OTX015 and AZD5153, have been developed to modulate BRD4 activity, and revert resistance to antiandrogens [18], with AZD5153 offering improved specificity and affinity due to its bivalent nature [19, 20]. Many BET inhibitors are in clinical trials for prostate cancer [16, 20], however, they face challenges related to toxicity, underscoring the need for dose reduction and possibly combination strategies [16, 19]. Preclinical evidence suggests that BET inhibitors may enhance the efficacy of external beam radiation therapy [17].

Therefore, in this study we set up to evaluate the potential of combining the PSMA-targeted alpha-emitting radioligand [^212^Pb]Pb-AB001 with BET inhibitors (AZD5153 or JQ1) in 2D monolayer cultures and 3D tumour spheroids to ultimately enhance therapeutic efficacy for mCRPC patients. Notably, [^212^Pb]Pb-AB001 is currently under clinical investigation for mCRPC, with a recent Phase 0 study confirming its safety, in vivo stability and favourable biodistribution [21]. The BET inhibitors JQ1 and AZD5153 were selected based on their demonstrated antitumor activity in prostate cancer models [13, 22–25]. AZD5153, a bivalent BET inhibitor with optimized pharmacokinetics and oral bioavailability, is currently under investigation in multiple phase I/II clinical trials for solid tumours, including mCRPC (e.g., NCT03205176) [33]. In parallel, JQ1 a monovalent BET inhibitor, extensively used in preclinical research, was included as a mechanistic comparator.

## Methods

### Cell culture

Human prostate cancer cell line C4-2 (ATCC CRL-3314, Manassas, VA) was subcultured in RPMI-1640 medium (Sigma-Aldrich (Merck), Oslo, Norway) supplemented with 10% heat-inactivated fetal bovine serum (FBS; Cytiva, Marlborough, MA), 100 units/mL penicillin and 10 µg/mL streptomycin (Sigma-Aldrich) and maintained at 5% CO_2_, 37 °C in a humidified incubator. Routine testing for mycoplasma was performed using MycoAlert Mycoplasma Detection kit (Lonza, Basel, Switzerland).

### BET inhibitors

The BET inhibitors AZD5153 (Cayman Chemical, Ann Arbor, MI) and JQ1 (MedChemExpress, NJ) were prepared in dimethyl sulfoxide (DMSO; Sigma-Aldrich) at a stock concentration of 25 mM and stored at −20 °C.

### Preparation of [^212^Pb]Pb-AB001

High purity ^212^Pb (≥ 99.99%) was generated by ^228^Th-based gas-diffusion generator and extracted in 0.1 M HCl, as described by Li et al. [26]. Prior to radiolabelling, the ^212^PbCl_2_ solution was pH-adjusted to 5–6 using 5 M sodium acetate. The AB001 ligand (previously referred to as NG001, MedKoo Biosciences Inc., Morrisville, NC, USA) was then added to achieve a specific activity of 2 MBq/µg (3.32 MBq/nmol), following the procedure described by Stenberg et al. [27]. The radioactivity of ^212^Pb in equilibrium with its progenies was measured using Cobra II Autogamma Counter (Packard Instrument Company, Downer Grove, IL) with a counting window 50–120 keV and Capintec CRC-127R radioisotope dose calibrator (Capintec Inc., Ramsey, NJ) with calibration number 667 [28]. Radiochemical purity was assessed using instant thin-layer chromatography (iTLC) with Tec-Control chromatography strips (Biodex Medical Systems, Shirley, NY, USA). Only radioligand preparations demonstrating a purity greater than 95% were used in the experiments.

### Treatments and assessment of metabolic activity in 2D cell monolayer

Cells were seeded in 96-well plates one day prior to initiating treatment. For single therapy treatments, the culture medium was replaced with either medium containing BET inhibitors for the entire treatment duration or medium containing [^212^Pb]Pb-AB001 for a 4-h period, followed by replacement of the radioligand with fresh medium. For combination therapy treatments, medium containing BET inhibitors was added 3–4 h prior to a 4-h incubation with [^212^Pb]Pb-AB001, after which, radioligand-containing medium was replaced with medium containing BET inhibitors.

For the C4-2 cell line, which expresses PSMA with a dissociation constant of approximately 22 nM for the ligand [29], the maximum radioactivity concentration of 50 kBq/mL was chosen. Given a specific activity of 2 MBq/µg, this corresponds to a ligand concentration of ~ 25 ng/mL, or ~ 15 nM — remaining below 22 nM ensures that the majority of PSMA receptors remain unoccupied by cold ligand, thereby allowing the observed cytotoxic effects to be attributed primarily to the radiolabelled compound rather than competitive binding by excess unlabelled AB001.

Metabolic cell activity, based on ATP quantification, was assessed using the CellTiter-Glo™ Assay (Promega, Madison, WI, USA) following the manufacturer’s protocol. Luminescence, proportional to intracellular ATP levels, was measured in clear-bottomed white plates with an integration time of 1000 ms using a Tecan Spark multimode microplate reader (Tecan, Mannedorf, Switzerland).

### Assessment of cytotoxicity in 3D spheroid model

C4-2 spheroids were generated by the liquid-overlay technique as described by Stenberg et al. [30]. Briefly, 96-well flat-bottomed plates were coated with a layer of 1.5% agarose in PBS with Mg^2+^ & Ca^2+^. C4-2 cells (500 cells/100 µL) were then added, and plates were centrifuged at 470 × g for 15 min. Spheroids formed over 4–5 days at 5% CO_2_, 37˚C in a humidified incubator. Spheroids were treated with drugs as described above.

Cell viability in spheroids was assessed using fluorescein diacetate (FDA; 16 µg/mL in PBS with Mg^2+^ & Ca^2+^) for live cells and propidium iodide (PI; 40 µg/mL in PBS with Mg^2+^ & Ca^2+^) for dead cells.

Spheroid growth and viability were monitored for up to 21 days using Axiovert 200 M microscope (Carl Zeiss AG, Oberkochen, Baden-Wurttemberg, Germany) and AxioVision Rel. 4.8 software (Carl Zeiss AG), with medium changes 2–3 times a week.

Spheroid size was assessed by measuring the cross-sectional area of each spheroid from brightfield images acquired at defined time points. Growth kinetics were assessed based on area changes over time. Doubling time (T_d_) was calculated by fitting the exponential growth phase using SigmaPlot15 to obtain the growth rate constant (a), with T_d_ = ln(2)/a.

### Evaluation of cell viability, DNA damage and cell cycle changes

C4-2 cells (0.5–2 × 10^6^ cells/flask) were seeded in T25 or T75 cell culture flasks one day before treatment. Cells were treated with drugs as described above. Cell death, DNA damage, cell cycle changes and the percentage of mitotic cells were assessed by harvesting cells at 1 h (only groups treated with [^212^Pb]Pb-AB001), 1, 3 and 6 days post-treatment.

Cell viability was assessed immediately post harvesting by staining with Annexin V FITC (ImmunoTools, Friesoythe, Germany) and PI (Sigma-Aldrich Norway AS) in Annexin V binding buffer (10 mM HEPES, 140 mM NaCl and 2.5 mM CaCl_2_) for 15 min at room temperature.

DNA damage and cell cycle changes were assessed by staining cells with FVD-eFluor 450 (cat. no. 65–0863-14, Invitrogen, Thermo Fisher Scientific, Oslo, Norway) for 1 h on ice, followed by fixation with 100% methanol (Sigma-Aldrich). Washes were performed with 0.2% v/v Tween-20 in PBS without Mg^2+^ & Ca^2+^ (PBST). Cells were stained with 1 µg/mL rabbit anti-PS10H3 (cat. no. 06–570, Merck Millipore, Darmstadt, Germany) and 1 µg/mL mouse anti-pγH2AX (cat. no. 05–636, Merck Millipore) primary antibodies in PBST with 2% v/v FBS and 0.4 mg/mL PureLink RNase A (cat. no. 12091–021, Invitrogen, Thermo Fisher Scientific) at room temperature, in the dark for 1 h. After removing the primary antibodies, cells were incubated with 2 µg/mL donkey anti-rabbit AlexaFluor647 (cat. no. A31573, Invitrogen, Thermo Fisher Scientific) and 2 µg/mL goat anti-mouse FITC (cat. no. F0479, Dako, Agilent Technologies, Santa Clara, CA, USA) secondary antibodies in PBST with 2% v/v FBS at room temperature in the dark for 30 min. Finally, cells were incubated with 10 µg/mL PI in PBST. Samples were then measured using a CytoFlex S Flow Cytometer (Beckman Coulter, Inc., Brea, CA, USA) with C Flow Plus Software and the data analysed using FlowJo 10.7.1 software (FlowJo LLC, Ashland, OR, USA).

### Statistical analysis

Data are presented as mean ± standard deviation. Statistical analyses were conducted using SigmaPlot 15.0 software (Systat Software, Inc., San Jose, CA, USA) and differences were deemed statistically significant at *p*-values < 0.05. The statistical difference between groups treated with [^212^Pb]Pb-AB001 versus combination treatment was evaluated using Student’s t-test when both normality (Shapiro–Wilk test) and equal variance assumptions were satisfied; otherwise, non-parametric alternatives (Mann–Whitney U test) were applied. For comparisons involving more than two groups, one-way ANOVA followed by Holm–Šídák post hoc correction for multiple comparisons was used, where applicable.

The Bliss independence test was performed to evaluate the presence of synergy between the two therapies [31]. It was assumed that both BET inhibitors and [^212^Pb]Pb-AB001 had independent effects, allowing for a comparison between the expected additive values and the observed values. The statistical significance of the difference between the calculated additive and observed values was assessed using the Student’s t-test, provided the normality and equal variance assumptions were met. Statistical significance was considered at *p* < 0.05. The combination index (CI) values were calculated from the mean values of two or three independent experiments using CompuSyn software (Combosyn, Inc., Paramus, NJ, USA). Interpretation of CI values followed the criteria: CI > 1 indicated antagonism, CI ≈ 1 indicated an additive effect, and CI < 1 indicated synergy [32].

All exact p-values are provided in Supplementary Tables S1–S5.

## Results

### Cytotoxicity of treatments with [^212^Pb]Pb-AB001 and BET inhibitors alone and in combination in 2D models

**Fig. 1:**
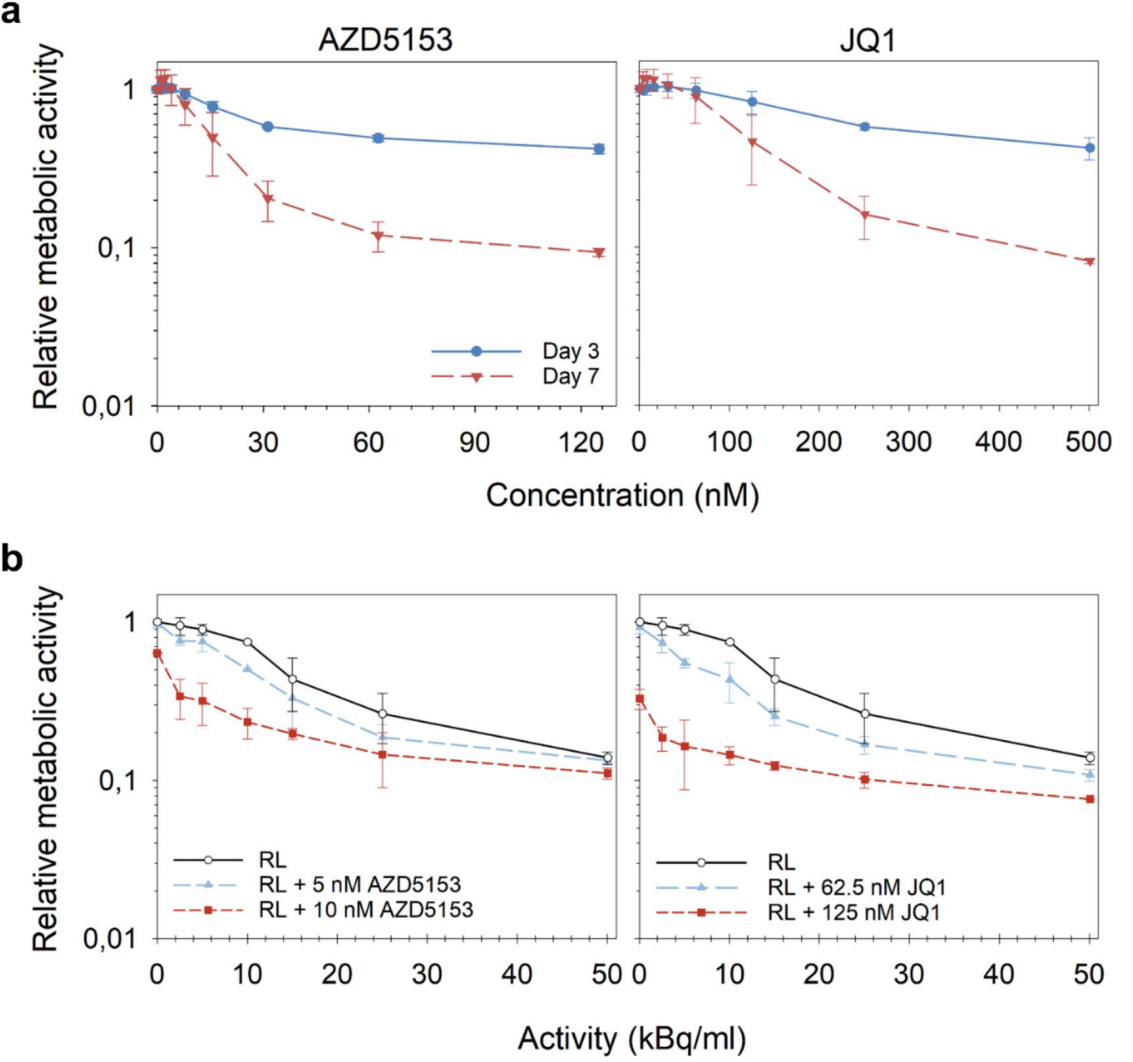
Effects of [^212^Pb]Pb-AB001 and BET inhibitors alone and in combination on metabolic activity in 2D models. **a** C4-2 cells were treated with AZD5153 and JQ1 alone for 3 or 7 days continuously. **b** For radioligand treatment, [^212^Pb]Pb-AB001 (RL) was added for 4 h, followed by media replacement. For combination treatment, cells were pre-treated with BET inhibitors for 3–4 h, followed by 4-h RL exposure, and then maintained in BET inhibitor-containing medium for 7 days. The metabolic cell activity was assessed using the CellTiter-Glo® Luminescent Cell Viability assay, and the results are presented as relative luminescence units normalized to the untreated control group (set to 1). Data are presented as a mean of three independent experiments, each performed in triplicate

To examine the cytotoxicity of [^212^Pb]Pb-AB001 and BET inhibitors as monotherapies and in combination, C4-2 prostate cancer cells were first cultured in monolayer. Both BET inhibitors, AZD5153 and JQ1, induced activity- and time-dependent reduction in relative metabolic cell activity (Fig. 1a). AZD5153 demonstrated significantly greater potency compared with JQ1, achieving a ≥ ninefold reduction in metabolic activity at equivalent concentrations (Fig. 1a). The half-maximal inhibitory concentrations (IC_50_) at day 7 were determined to be 16 nM for AZD5153 and 130 nM for JQ1 (Fig. 1a, Supplementary Table S1).

Combination treatments significantly reduced metabolic activity in C4-2 cells compared to [^212^Pb]Pb-AB001 monotherapy (*p* < 0.05, Fig. 1b, Supplementary Table S3). While [^212^Pb]Pb-AB001 alone required an activity of ~ 14 kBq/mL to reach IC_50_, co-treatment with 10 nM AZD5153 or 62.5 nM JQ1 lowered this value to 2 kBq/mL and 7 kBq/mL, respectively (Fig. 1b, Supplementary Tables S1 and S2). The CI values calculated on day 7 indicated additive effects (Table 1; CI ≈ 1).

**Table 1:** Combination Index (CI) values calculated at 7 days post-treatment. CI values were calculated using CompuSyn software, based on data in Fig. 1. CI < 1 indicates synergism, CI = 1 additivity, and CI > 1 suggests antagonism

[^212^Pb]Pb-AB001 (kBq/mL)	CI			
	AZD5153		JQ1	
	5 nM	10 nM	62.5 nM	125 nM
2.5	0.8	0.8	1.0	0.9
5	1.1	0.8	1.0	0.9
10	1.0	0.9	1.1	1.0
15	1.0	1.0	1.0	1.1
25	1.0	1.1	1.0	1.2
50	1.3	1.4	1.3	1.4

### Growth inhibition of [^212^Pb]Pb-AB001 and BET inhibitors in 3D models

**Fig. 2:**
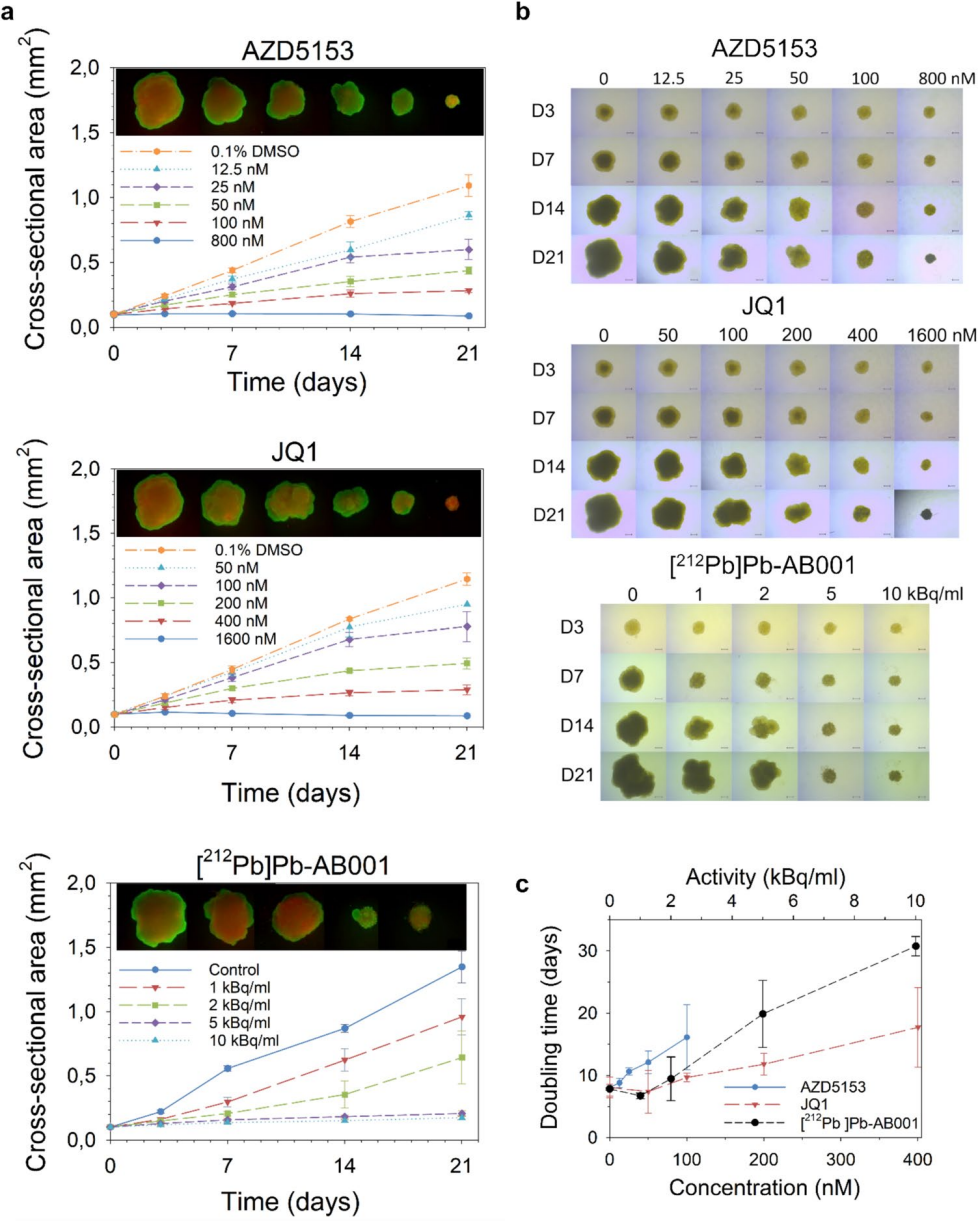
Effects of [^212^Pb]Pb-AB001 and BET inhibitors on growth and viability of C4-2 spheroids. C4-2 spheroids were treated with AZD5153 or JQ1 continuously for 21 days. For radioligand treatment, [^212^Pb]Pb-AB001 was administered for 4 h, followed by medium replacement. For combination treatment, spheroids were pre-treated with BET inhibitors for 3–4 h, exposed to [^212^Pb]Pb-AB001 for 4 h, and then maintained in medium containing the respective BET inhibitor for the remainder of the 21-day period. **a** Cross-sectional area measurements of C4-2 spheroids over time, **b** Representative images and **c** the doubling times of treated spheroids. Images were captured using 4 × magnification, with a scale bar of 200 µm. The images shown are representative of a single experiment. Live–dead staining was performed using fluorescein diacetate (FDA) and propidium iodide (PI). FDA is a membrane-permeable dye that is hydrolyzed by intracellular esterases in viable cells to produce green fluorescence. PI is membrane-impermeable and intercalates into DNA of membrane-compromised (dead or dying) cells, emitting red fluorescence. Spheroids were stained on day 21 post-treatment to assess viability distribution

**Fig. 3:**
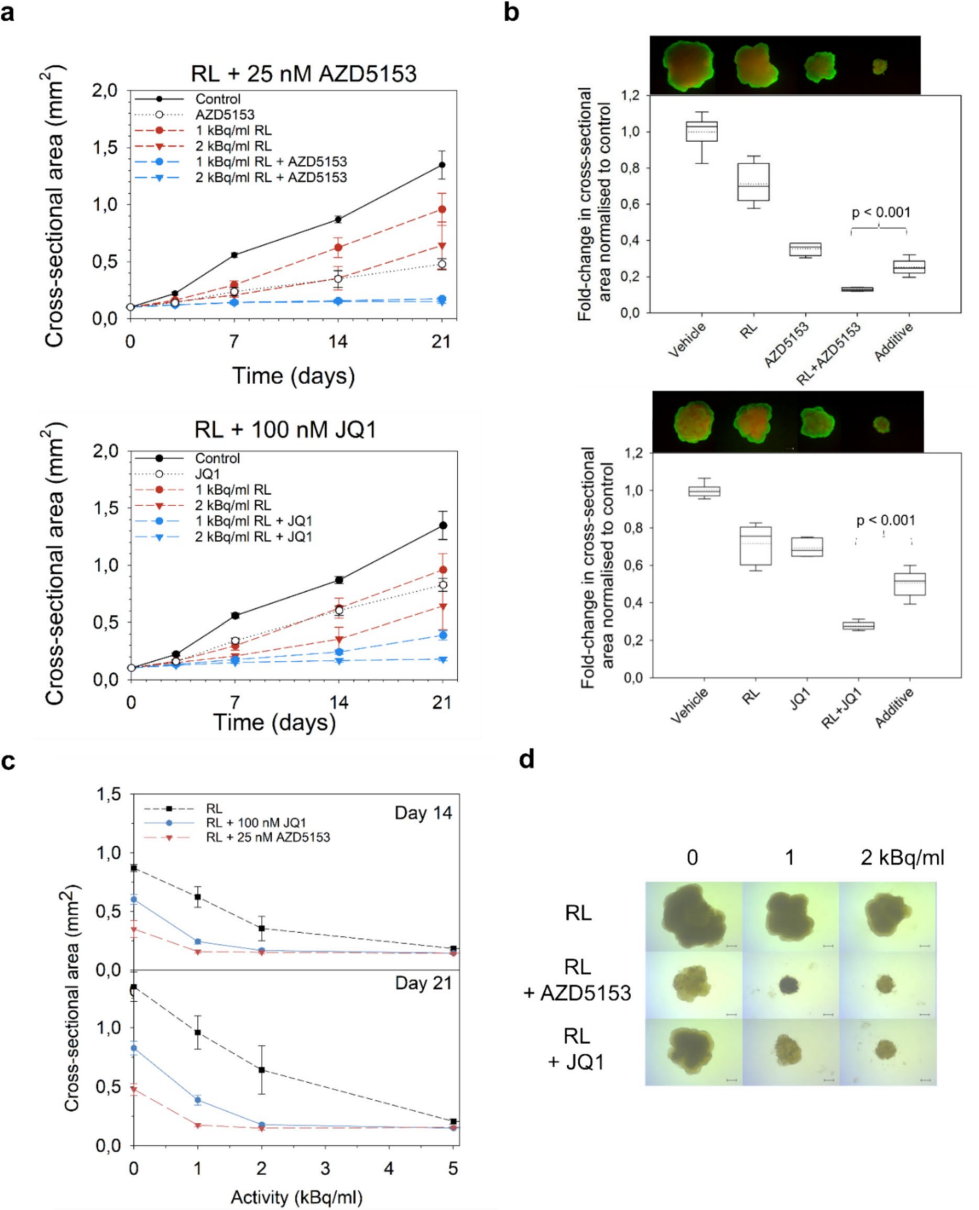
Effects of [^212^Pb]Pb-AB001 combination with BET inhibitors on growth and viability of C4-2 spheroids. C4-2 spheroids were treated with AZD5153 or JQ1 continuously for 21 days. For radioligand treatment, [^212^Pb]Pb-AB001 was administered for 4 h, followed by medium replacement. For combination treatment, spheroids were pre-treated with BET inhibitors for 3 – 4 h, exposed to [^212^Pb]Pb-AB001 for 4 h, and then maintained in medium containing the respective BET inhibitor for the remainder of the 21-day period. **a** Cross-sectional area measurements of C4-2 spheroids over time following treatment with [^212^Pb]Pb-AB001 (RL), AZD5153 or JQ1 alone and in combination. **b** Fold-change in spheroid cross-sectional area, normalized to control, after combination treatments of 25 nM AZD5153 with 1 kBq/mL [^212^Pb]Pb-AB001 on day 21 (top) and 100 nM JQ1 with 2 kBq/mL [^212^Pb]Pb-AB001 on day 14 (bottom), with vehicles representing untreated controls, RL = treatment with [^212^Pb]Pb-AB001, AZD5153 or JQ1 for BET inhibitors, RL + AZD5153 or JQ1 for combination treatments, and "additive" for calculated theoretical additive effects according to the Bliss model. P-value indicates 2 -tailed t-test against the observed values for combination treatment. *p* < 0.05 indicates synergism. **c** Cross-sectional area plotted against activity for combination treatments on days 14 and 21 with [^212^Pb]Pb-AB001 alongside 25 nM AZD5153 or 100 nM JQ1. **d** Representative images of spheroid morphology captured on day 21. Live–dead staining was performed using fluorescein diacetate (FDA) and propidium iodide (PI). FDA is a membrane-permeable dye that is hydrolyzed by intracellular esterases in viable cells to produce green fluorescence. PI is membrane-impermeable and intercalates into DNA of membrane-compromised (dead or dying) cells, emitting red fluorescence. Spheroids were stained on day 14 and 21 post-treatment to assess viability distribution

The effects of [^212^Pb]Pb-AB001, BET inhibitors (AZD5153 or JQ1), and their combinations were assessed for up to 21 days using a C4-2 spheroid model (Figs. 2 and 3). Each treatment alone demonstrated an activity or concentration-dependent inhibition of spheroid growth (Fig. 2). AZD5153 and JQ1 suppressed spheroid growth with IC₅₀ values of ~ 50 nM and ~ 200 nM, respectively, and achieved complete growth arrest at 800 nM and 1600 nM, respectively (Fig. 2). These treatments significantly extended the doubling time from 8 ± 1 days in control spheroids to 16 ± 5 days with 100 nM AZD5153 and 18 ± 6 days with 400 nM JQ1 (Fig. 2c). The AZD5153 and JQ1 curves stop at 100 nM and 400 nM, respectively (Fig. 2c) because higher concentrations strongly inhibited spheroid growth, preventing reliable doubling time calculations.

[^212^Pb]Pb-AB001 exhibited activity-dependent growth inhibition, with an estimated IC_50_ of ~ 1.7 kBq/mL on both day 14 and day 21, indicating sustained cytotoxicity (Fig. 2a). Complete growth arrest was observed at 10 kBq/mL (Fig. 2a). Despite this, live–dead staining on day 21 revealed metabolically active cells within spheroids treated with the highest concentrations of BET inhibitors or [^212^Pb]Pb-AB001. (Fig. 2a).

For combination treatments, AZD5153 (12.5, 25, and 50 nM) and JQ1 (50, 100 and 200 nM) concentrations, approximating IC_10_, IC_25_, and IC_50_ values, were selected. Combined treatments with [^212^Pb]Pb-AB001 prevented spheroid regrowth at 1–2 kBq/mL, which was otherwise observed by day 14 following monotherapy (Fig. 3). Spheroid size analysis confirmed enhanced reduction with combinations compared to [^212^Pb]Pb-AB001 alone (Fig. 3c, Supplementary Table S4). FDA-PI staining in the spheroids indicated the presence of metabolically active cells following combination treatment with AZD5153 and JQ1 (Fig. 3b). Bliss independence analysis demonstrated synergistic effects of BET inhibitors and [^212^Pb]Pb-AB001 (Fig. 3b, Supplementary Table S5). CI values calculated at days 14 and 21 further supported synergy, particularly at lower radioligand activities (Table 2). AZD5153 showed stronger synergy, especially at 12.5 and 25 nM, with CI values consistently below 0.7 across all activity levels (Table 2).

**Table 2:** Combination Index (CI) values for C4-2 spheroids calculated after 14 and 21 days of treatment from two independent experiments each performed in sextuplicate using CompuSyn software. RL = [^212^Pb]Pb-AB001

BET inhibitors	Concentration (nM)	CI		
		1 kBq/mL RL	2 kBq/mL RL	5 kBq/mL RL
Day 14				
AZD5153	12.5	0.3	0.4	0.6
	25	0.5	0.4	0.7
	50	0.6	0.5	0.7
JQ1	50	0.9	0.5	0.7
	100	0.7	0.6	0.7
	200	0.6	0.6	0.9
Day 21				
AZD5153	12.5	0.5	0.3	0.5
	25	0.4	0.3	0.6
	50	0.5	0.4	0.6
JQ1	50	1.0	0.7	0.6
	100	0.8	0.7	0.6
	200	0.6	0.5	0.8

### Treatment combinations of [^212^Pb]Pb-AB001 and BET inhibitors alter cell cycle distribution and DNA damage accumulation

**Fig. 4:**
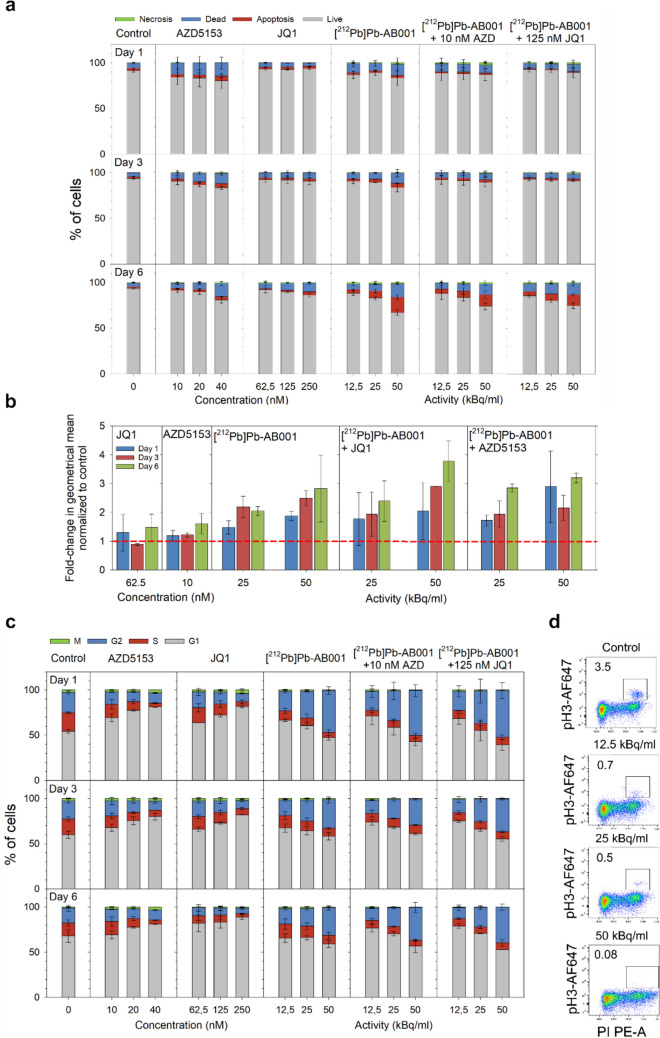
Effect of [^212^Pb]Pb-AB001 and BET inhibitors on cell cycle phase responses and DNA damage. C4-2 cells were treated with AZD5153 or JQ1 continuously for 1, 3 or 6 days. For radioligand treatment, [^212^Pb]Pb-AB001 was administered for 4 h, followed by medium replacement. For combination treatment, cells were pre-treated with BET inhibitors for 3–4 h, exposed to [^212^Pb]Pb-AB001 for 4 h, and then maintained in medium containing the respective BET inhibitor for the remainder of period. **a** Cell viability, **b** DNA damage and **c**, **d** cell cycle distribution in C4-2 cells post-treatment were analysed by flow cytometry. **a** Viability assessed using Annexin V FITC and propidium iodide (PI) staining on days 1, 3 and 6 to distinguish viable, apoptotic and necrotic cells. **b** DNA damage was assessed by comparing the fold change in the geometrical mean fluorescence intensity of γH2AX-positive cells between untreated controls and treated groups at 1, 3, and 6 days post-treatment. The red line indicates the untreated control (**c**) Cell cycle distribution following continuous treatment with BET inhibitors (10 nM AZD5153 or 125 nM JQ1) and a 4-h exposure to [^212^Pb]Pb-AB001, individually and in combination. **d** Mitotic activity evaluated 1 h after a 4-h incubation with [^212^Pb]Pb-AB001. Data represent mean ± SD from three independent experiments

We further evaluated changes in cell death, cell cycle distribution and DNA damage accumulation in C4-2 cells using flow cytometry on days 1, 3 and 6 after 4-h exposure to [^212^Pb]Pb-AB001 and continued treatment with AZD5153 or JQ1 combination (Fig. 4).

BET inhibitors alone had limited effects on cell apoptosis, necrosis, and overall cell death, as assessed by Annexin V/PI flow cytometry (Fig. 4a). JQ1 did not significantly induce apoptosis and cell death, while AZD5153 at 40 nM induced a significant increase in number of dead cells by day 6 (14 ± 2%, *p* < 0.05), although apoptosis did not significantly increase, peaking at 5 ± 2% on day 1. In contrast, [^212^Pb]Pb-AB001 induced an activity and time-dependent significant increase in cell death, with the most significant effect observed on day 6 at 50 kBq/mL (17 ± 3%, *p* < 0.05). We did not observe significant increase in necrotic cells at any time point, indicating that cell death was predominantly apoptotic. The combination of [^212^Pb]Pb-AB001 with BET inhibitors did not enhance apoptotic cell death compared with [^212^Pb]Pb-AB001 alone, suggesting that the additive and synergistic effect observed in cytotoxicity experiments is not due to the augmented apoptosis.

BET inhibitors primarily induced G1-phase accumulation, particularly at higher concentrations and later time points (Fig. 4c). AZD5153 at 40 nM increased accumulation of cells in G1 from 54% (control) to 81% on day 1 (*p* < 0.05). JQ1 induced a similar effect at 250 nM. Both BET inhibitors led to depletion of cells in the S and G2 phases, indicating cell cycle arrest in G1. In contrast, treatment with [^212^Pb]Pb-AB001 alone induced activity-dependent cell cycle changes, primarily leading to G2-phase accumulation. At 50 kBq/mL, the G2 population increased from 22% (control) to 46% on day 1 (*p* < 0.05), followed by a decrease to 32% on day 3 and 30% on day 6, suggesting a transient G2 arrest. Accumulation in G2 was accompanied by a reduction in the S-phase population, which dropped from 21% (control) to 6% at 50 kBq/mL on day 1 (*p* < 0.05), with a further decline over time. [^212^Pb]Pb-AB001 also caused an activity-dependent reduction in mitotic cells (Fig. 4c, d), indicating that alpha-particle irradiation strongly inhibits cell cycle progression into mitosis, likely due to DNA damage-induced G2 arrest. BET inhibitors also reduced the mitotic cell population, further supporting their role in cell cycle inhibition (Fig. 4c).

Combination treatment with [^212^Pb]Pb-AB001 and BET inhibitors did not exhibit additive effects on G2-phase arrest, as [^212^Pb]Pb-AB001 alone was sufficient to induce pronounced G2 accumulation (Fig. 4c). Overall, the data suggest that [^212^Pb]Pb-AB001 primarily induces G2 arrest, while BET inhibitors arrest cell cycle in G1, with minimal combined effects on cell cycle progression.

[^212^Pb]Pb-AB001 exposure showed a trend toward an activity- and time-dependent increase in DNA damage, assessed via phosphorylated γH2AX, although this was not significant due to high variability between experiments (Fig. 4b). Fold-change of phosphorylated γH2AX compared to control increased from 1.9 on day 1 to 2.8 on day 6 at 50 kBq/mL. By day 6, DNA damage levels remained elevated, suggesting persistent DNA breaks and insufficient repair. BET inhibitors alone induced minimal DNA damage across all time points and combination treatments did not consistently enhance DNA damage beyond the effects of [^212^Pb]Pb-AB001 alone (Fig. 4b).

## Discussion

This study demonstrates that combining BET inhibitors with the PSMA-targeted alpha-emitting radioligand [^212^Pb]Pb-AB001 enhances cytotoxicity in prostate cancer cells, particularly in 3D tumour spheroids. While both monotherapies inhibited cancer cell growth, their combination produced significantly greater effects in 3D models, indicating therapeutic synergy that is not fully captured in conventional 2D assays. Among the two BET inhibitors tested, AZD5153 consistently outperformed JQ1 in both 2D and 3D settings, likely due to its bivalent binding to BRD4’s tandem bromodomains, resulting in more potent transcriptional repression of oncogenic targets such as MYC and AR.

AZD5153 at 40 nM increased the percentage of dead cells, including necrotic and apoptotic, indicating that BRD4 is a primary target in prostate cancer. Compared to JQ1, AZD5153 is more clinically advanced with therapeutic doses (10–30 mg/day) yielding peak plasma concentrations of approximately 50–250 nM [20]. Accordingly, the concentrations used in our combination studies (5–50 nM for AZD5153) lie within or below this clinically achievable range, reinforcing the translational relevance of our results.

The 3D spheroid model provided a more physiologically relevant platform for assessing treatment efficacy. All monotherapies suppressed spheroid growth to varying degrees, combination treatments led to more pronounced inhibition of regrowth and significantly prolonged spheroid doubling times. Notably, synergistic effects were observed, particularly at lower concentrations of AZD5153 and [^212^Pb]Pb-AB001 (CI < 1, Table 2). The enhanced efficacy of [^212^Pb]Pb-AB001 in 3D versus 2D cultures highlights the contributions of alpha-particle cross-fire effect. The estimated IC_50_ in spheroids was approximately 1.7 kBq/mL, compared to 14 kBq/mL in 2D monolayers, an ~ eightfold difference. This likely reflects enhanced energy deposition across densely packed cell layers in 3D architecture. Live-dead staining on day 21 revealed central necrosis with viable cells persisting at the periphery, suggesting that although combination therapy effectively suppresses regrowth, complete spheroid eradication may require prolonged exposure or higher cumulative doses. These findings underscore the value of 3D models in capturing combinatorial effects that may be underestimated in monolayer systems.

In 2D assays, [^212^Pb]Pb-AB001 induced a delayed apoptotic response and marked G2-phase arrest, consistent with the well-established radiobiological effects of high-LET alpha particles in generating complex, difficult-to-repair DNA double-strand breaks [33–35]. In contrast, BET inhibitors primary caused G1-phase arrest and reduced proliferation. Although AZD5153 modestly increased cell death at higher concentrations, neither BET inhibitor significantly enhanced [^212^Pb]Pb-AB001-induced DNA damage, G2 arrest, mitotic arrest or apoptosis. These findings suggest that synergy observed in 3D models is driven more by durable proliferative arrest than by early cytotoxic effects.

Cell cycle analyses further elucidated the mechanistic divergence between the two treatment modalities. BET inhibitors induced a G1-phase arrest (Fig. 4c), consistent with their known ability to suppress transcription of key cell cycle regulators, such as MYC and E2F, resulting in reduced entry into S-phase and a cytostatic phenotype [13, 36, 37]. In contrast, [^212^Pb]Pb-AB001 caused G2-phase accumulation, reflecting activation of the DNA damage response to high-LET alpha radiation and engagement of the G2/M checkpoint to allow for double-strand break repair [38, 39]. The lack of enhanced G2 arrest in the combination-treated cells likely results from BET inhibitor-induced G1 arrest, which limits the number of cells progressing to S and G2 phases. This indicates that the agents act through distinct mechanisms and at different checkpoints. Rather than synergizing at the level of G2 checkpoint activation, the treatments likely exert complementary effects, BET inhibitors halting proliferation and [^212^Pb]Pb-AB001 inducing irreparable DNA damage. This mechanistic divergence may explain why combination treatment did not enhance early apoptosis (Fig. 4a), as measured by Annexin V/PI staining.

Supporting this hypothesis, spheroids treated with the highest combination doses exhibited complete growth arrest without visible shrinkage or disintegration over 21 days, suggesting a long-term cytostatic state rather than rapid induction of apoptosis. This effect is consistent with the sustained activation of the G2/M checkpoint following high-LET radiation, as cells attempt to repair complex DNA lesions prior to mitosis [8, 40]. Because BET inhibitors prevent progression into S and G2 phases, they limit the capacity of cells to respond to alpha-induced DNA damage by stalling them in G1, thus modulating the damage response without converging on a shared cell cycle arrest point. Ultimately, the combined treatment suppresses tumour cell regrowth through complementary, non-overlapping mechanisms, transcriptional silencing and irreversible genomic insult, resulting in long-term growth inhibition.

Although [^212^Pb]Pb-AB001 induced DNA damage in 2D monolayers, the addition of BET inhibitors did not lead to increase in γH2AX levels as measured by flow cytometry, suggesting no measurable enhancement in DNA double-strand break accumulation. This may be due to BET inhibitor-mediated suppression of DNA repair genes, rather than enhanced formation of new breaks. BET inhibitors alone induced minimal DNA damage [15], but previous studies have shown that they disrupt the homologous recombination repair pathway by downregulating BRCA1, RAD51, CtIP, and other repair proteins through transcriptional repression via BRD2/3/4 inhibition [41]. BET inhibitors also downregulate additional DNA repair regulators such as BRCA2, CHK1/CHK2, MRE11, and WEE1, thereby compromising multiple components of the DNA damage response network [41]. Moreover, BET inhibition promotes transcription-associated R-loop accumulation and endogenous double-strand breaks, sensitizing cells to additional DNA-damaging agents such as alpha radiation. While we did not directly quantify HR protein expression in this study, our functional data, persistent DNA damage, G2-phase arrest, and enhanced cytostatic effects, are consistent with BET-induced impairment of DNA repair. Future work should include transcriptomic or proteomic profiling to validate suppression of HR and elucidate how this contributes to increased sensitivity to alpha radiation. This mechanistic insight will inform rational design of combination regimens and support clinical translation.

Although synergistic effects were observed in 3D spheroids, no significant increase in apoptosis was detected in the combination groups in 2D cultures following combination treatments. Several factors may explain this discrepancy. BET inhibitors, such as AZD5153 and JQ1, primarily induce G1-phase arrest and reduce proliferation without directly activating apoptotic pathways [42, 43]. In our study, AZD5153 reduced proliferation but induced only modest induction of apoptosis, both as monotherapy and in combination with [^212^Pb]Pb-AB001. These findings suggest that the observed synergy is largely driven by durable growth suppression rather than acute cytotoxicity. Moreover, the 6-day experimental window used for apoptosis detection may not fully capture the kinetics of delayed cell death, which is known to occur following alpha-particle-induced DNA damage. Supporting this, spheroids treated with the highest drug and activity combinations showed complete growth arrest without visible shrinkage or disintegration over 21 days. This indicates a state of long-term proliferation arrest rather than active cell death. Therefore, the therapeutic benefit may reflect cumulative cytostatic effects or induction of senescence rather than apoptosis or mitotic catastrophe within the timeframe evaluated. Future studies should include extended follow-up, clonogenic survival assays, or senescence markers to more comprehensively assess long-term treatment outcomes.

While extensive research has been conducted on BET inhibitors and targeted radionuclide therapy individually, studies specifically investigating their combination remain scarce. Most current efforts focus on combining BET inhibitors with chemotherapy, epigenetic drugs or molecularly targeted agents [44, 45]. For example, a phase Ib/IIa study demonstrated that the combination of the BET inhibitor ZEN-3694 and the androgen receptor inhibitor enzalutamide was well tolerated and showed promising clinical activity in patients with enzalutamide- or abiraterone-resistant mCRPC, particularly in those with low androgen receptor transcriptional activity [46]. Targeted alpha radioligand therapy offers a compelling strategy to overcome resistance in such cases by delivering potent, localized cytotoxic radiation independent of androgen receptor signalling. Our study addresses a critical gap in this field, providing the first evidence of synergy between BET inhibition and alpha-emitting radioligands in PSMA-expressing prostate cancer cells.

Several limitations should be considered when interpreting the findings in this study. First, this study utilized only one prostate cancer cell line, C4-2, which may not fully capture the heterogeneity mCRPC. C4-2 cells were selected based on their endogenous PSMA expression and robustness under sequential treatment conditions. Other prostate cancer cell lines were evaluated but found unsuitable for this study: DU-145 and PC-3 lack PSMA expression, PC-3 PIP cells express artificially high levels of PSMA, and LNCaP cells showed poor tolerance to the experimental procedures, resulting in substantial cell loss. Future studies should evaluate multiple prostate cancer models with varying genetic backgrounds to determine whether specific mutations influence the efficacy of combination therapy. Second, while the 3D spheroid model provides a more physiologically relevant system compared to 2D cultures, it still does not fully replicate the tumour microenvironment, particularly in terms of immune interactions and vascularization. The current study was designed as a focused in vitro investigation to establish mechanistic rationale and evaluate the combinatorial efficacy of [^212^Pb]Pb-AB001 and BET inhibitors prior to advancing to in vivo models. The observed PSMA-dependent cytotoxicity, enhanced cell cycle arrest, and synergistic growth suppression in spheroids provide a strong preclinical foundation. In vivo studies using PSMA-positive xenograft models will be essential to determine whether the therapeutic effects observed in spheroids translate into improved treatment outcomes in a more clinically relevant context.

## Conclusions

This study demonstrates that the combination of the BET inhibitor AZD5153 with the PSMA-targeted alpha-emitting radioligand [^212^Pb]Pb-AB001 enhances therapeutic efficacy in prostate cancer models, with synergistic effects observed specifically in 3D spheroids. While both agents suppressed cancer cell growth as monotherapies, their combination led to significantly greater inhibition of spheroid regrowth and prolonged doubling times, particularly at lower concentrations, supporting the presence of therapeutic synergy in a spatially organized tumour model. The cytotoxic effects of [^212^Pb]Pb-AB001 in 2D cultures were associated with delayed apoptosis and marked G2-phase arrest, consistent with the DNA damage response to high-LET alpha radiation. AZD5153 induced G1-phase arrest and reduced proliferation but caused only modest cell death. Combination treatment did not enhance early apoptotic response or G2-phase accumulation in 2D, suggesting that synergy in 3D models may arise from long-term growth suppression rather than immediate cytotoxicity. These findings highlight the added value of 3D tumour models in capturing treatment effects that may be underestimated in conventional monolayer systems. The concentrations of AZD5153 used in this study were within clinically relevant ranges, supporting the translational potential of this combination. Overall, the data establish a strong in vitro foundation for further investigation of combined BET inhibition and targeted alpha therapy in more complex models, including in vivo studies of PSMA-expressing prostate cancer.

## Supplementary Information

Below is the link to the electronic supplementary material.

Supplementary file1 (DOCX 791 KB)

## Data Availability

No datasets were generated or analysed during the current study.
