# Supplementary material for: Combination of PSMA targeting alpha-emitting radioligand [212Pb]Pb-AB001 with BET bromodomain inhibitors in in vitro prostate cancer models
Source: Med Oncol. 2025 Jul 22;42(8):362. doi: 10.1007/s12032-025-02925-9 (PMC12283873; doi:10.1007/s12032-025-02925-9)
Supplement: Supplementary file 1 — Supplementary file1 (DOCX 791 KB) [file 12032_2025_2925_MOESM1_ESM.docx]

**Combination of PSMA targeting alpha-emitting radioligand [^212^Pb]Pb-AB001** **with BET bromodomain inhibitors in *in vitro* prostate cancer models**

Rugile Liukaityte^1^, Vilde Yuli Stenberg^2^, Andrius Kleinauskas^1,3^, Petras Juzenas^1^, Alfonso Urbanucci^4,5^, Asta Juzeniene^1,5*^

^1^Department of Radiation Biology, Institute for Cancer Research, The Norwegian Radium Hospital, Oslo University Hospital, Oslo, Norway

^2^ARTBIO AS, Oslo, Norway

^3^Department of Physics, University of Oslo, Oslo, Norway

^4^Department of Tumour Biology, Institute for Cancer Research, The Norwegian Radium Hospital, Oslo University Hospital, Oslo, Norway

^5^Faculty of Medicine and Health Technology, Tampere University, TAYS Cancer Centre and FICAN Mid, Tampere, Finland

^*^Correspondence: Asta Juzeniene [astaj@ous-hf.no](mailto:astaj@ous-hf.no)

Rugile Liukaityte rugliu@ous-hf.no

Vilde Yuli Stenberg vilde.stenberg@artbio.com

Andrius Kleinauskas andrkl@ous-hf.no

Petras Juzenas petrasj@ous-hf.no

Alfonso Urbanucci alfonsourbanucci@gmail.com

[ORCID](https://orcid.org/) of the authors:

Rugile Liukaityte <https://orcid.org/0009-0009-7547-3287>

Vilde Yuli Stenberg <https://orcid.org/0000-0003-4329-5411>

Andrius Kleinauskas <https://orcid.org/0000-0002-6679-6705>

Petras Juzenas <https://orcid.org/0000-0002-0114-903X>

Alfonso Urbanucci <https://orcid.org/0000-0003-2931-3652>

Asta Juzeniene <https://orcid.org/0000-0001-9426-0062>

**Supplementary Materials**

**Supplementary Table 1.** IC_50_ values post 7-day continuous treatment with AZD5153 and JQ1 or 7 days post 4-hour treatment with [^212^Pb]Pb-AB001 (n=3 (BET inhibitors) and n=2 ([^212^Pb]Pb-AB001) independent experiments with three parallels for each group)

| IC | AZD5153 (nM) | JQ1 (nM) | [^212^Pb]Pb-AB001 (kBq/mL) |
| --- | --- | --- | --- |
| 10 | 4 ± 3 | 39 ± 20 | 3 ± 0 |
| 25 | 8 ± 3 | 66 ± 28 | 6 ± 0 |
| 50 | 16 ± 6 | 130 ± 47 | 14 ± 1 |
| 75 | 33 ± 10 | 250 ± 84 | 33 ± 4 |
| 90 | 61 ± 12 | 448 ± 138 | 75 ± 13 |

**Supplementary Table 2.** IC_50_ values for [^212^Pb]Pb-AB001 (kBq/mL) after combination with 5 – 10 nM AZD5153 or 62.5 – 125 nM JQ1 (n=2 (10 nM AZD5153 and 62.5 – 125 nM JQ1) and n=1 (5 nM AZD5153) independent experiments with three parallels for each group).

| IC | AZD5153 (nM) | | JQ1 (nM) | |
| --- | --- | --- | --- | --- |
|  | 5 | 10 | 62.5 | 125 |
| 10 | 0.7 | N/A | N/A | N/A |
| 25 | 4 | N/A | 3 ± 1 | N/A |
| 50 | 10 | 2 ± 1 | 7 ± 0 | N/A |
| 75 | 26 | 7 ± 6 | 19 ± 0.7 | 2 ± 1 |
| 90 | 76 | 140 ± 28 | 65 ± 4 | 18 ± 11 |

**Supplementary Table 3**. The summary of p-values for the relative metabolic viability assessment after treatment with a combination of BET inhibitors with [^212^Pb]Pb-AB001. For each group normality (Shapiro-Wilk test) and equal variance (Brown-Forsythe test) were assessed before proceeding with Student’s t-test if data was normally distributed or the non-parametric Mann-Whitney Rank Sum Test, if not. Grey fields indicate the Mann-Whitney Rank Sum Test. Statistically significant values are indicated in blue.

|  |  | [^212^Pb]Pb-AB001 (kBq/mL) | | | | | |
| --- | --- | --- | --- | --- | --- | --- | --- |
|  |  | 2.5 | 5 | 10 | 15 | 25 | 50 |
| AZD5153 (nM) | 5 | 0,083 | 0,263 | 0,002 | 0,296 | 0,163 | 0,711 |
|  | 10 | <0,001 | 4,12E-06 | 2,91E-09 | <0,001 | 0,006 | 0,006 |
| JQ1 (nM) | 62.5 | 0,020 | 6,64E-04 | 2,65E-05 | 0,010 | 0,016 | 0,004 |
|  | 125 | <0,001 | 9,3E-08 | <0,001 | <0,001 | 2,49E-04 | 5,59E-06 |

**Supplementary table 4.** The summary of p-values comparing combination of [^212^Pb]Pb-AB001 with AZD5153/JQ1 versus [^212^Pb]Pb-AB001 treatment alone in the 3D spheroid model. For each group normality (Shapiro-Wilk test) and equal variance (Brown-Forsythe test) were assessed before proceeding with Student’s t-test if data was normally distributed or the non-parametric Mann-Whitney Rank Sum Test, if not. Grey fields indicate the Mann-Whitney Rank Sum Test. Statistically significant values are indicated in blue.

| BETi | Concentration (nM) | Day | [^212^Pb]Pb-AB001 (kBq/mL) | | |
| --- | --- | --- | --- | --- | --- |
|  |  |  | 1 | 2 | 5 |
| AZD5153 | 12.5 | 3 | 7,35E-09 | 1,50E-04 | 0,177 |
|  |  | 7 | <0,001 | 1,81E-07 | 7,44E-05 |
|  |  | 14 | 7,23E-14 | 4,20E-05 | 3,00E-06 |
|  |  | 21 | 1,74E-08 | 2,41E-05 | <0,001 |
|  | 25 | 3 | 1,05E-14 | 2,63E-10 | 3,50E-04 |
|  |  | 7 | <0,001 | 7,29E-14 | 7,00E-09 |
|  |  | 14 | 1,45E-18 | 1,67E-08 | 2,29E-09 |
|  |  | 21 | 7,43E-14 | 1,18E-08 | <0,001 |
|  | 50 | 3 | 7,71E-14 | 8,91E-14 | 1,37E-07 |
|  |  | 7 | <0,001 | 1,69E-12 | 1,98E-09 |
|  |  | 14 | 3,73E-15 | 4,08E-06 | 6,15E-09 |
|  |  | 21 | 2,54E-11 | 6,74E-06 | 0,002 |
| JQ1 | 50 | 3 | 8,58E-05 | 0,002 | 2,85E-05 |
|  |  | 7 | <0,001 | 6,84E-05 | 0,122 |
|  |  | 14 | 0,001 | 0,002 | 0,003 |
|  |  | 21 | 0,018 | 0,010 | 0,016 |
|  | 100 | 3 | 2,09E-11 | 7,51E-09 | 0,003 |
|  |  | 7 | 1,94E-12 | 2,6E-10 | 1,17E-05 |
|  |  | 14 | 5,49E-11 | 1,35E-05 | 7,83E-05 |
|  |  | 21 | <0,001 | 8,07E-05 | 0,002 |
|  | 200 | 3 | 1,66E-09 | 4,23E-10 | 0,077 |
|  |  | 7 | 0,002 | 6,98E-10 | 1,39E-04 |
|  |  | 14 | 1,57E-11 | 6,29E-05 | 5,03E-04 |
|  |  | 21 | 4,89E-08 | 7,79E-05 | 0,022 |

**Supplementary table 5.** The summary of p-values of when comparing the calculated additive to the observed effect after combination of [^212^Pb]Pb-AB001 with AZD5153/JQ1 in the spheroid model. The p-values for 5 kBq/mL group are not included in the table due to small spheroid size resulting in high relative variation. For each group normality (Shapiro-Wilk test) and equal variance (Brown-Forsythe test) were assessed before proceeding with Student’s t-test if data was normally distributed or the non-parametric Mann-Whitney Rank Sum Test, if not. Grey fields indicate the Mann-Whitney Rank Sum Test. Statistically significant values are indicated in blue.

| BETi | Concentration (nM) | Day | [^212^Pb]Pb-AB001 (kBq/mL) | |
| --- | --- | --- | --- | --- |
|  |  |  | 1 | 2 |
| AZD5153 | 12.5 | 3 | 0,014 | 1,02E-10 |
|  |  | 7 | 0,003 | 0,021 |
|  |  | 14 | 2,16E-16 | 0,002 |
|  |  | 21 | <0,001 | 9,47E-05 |
|  | 25 | 3 | <0,001 | <0,001 |
|  |  | 7 | 0,034 | <0,001 |
|  |  | 14 | <0,001 | 0,055 |
|  |  | 21 | <0,001 | 6,42E-06 |
|  | 50 | 3 | 2,51E-06 | 8,98E-06 |
|  |  | 7 | <0,001 | 3,06E-17 |
|  |  | 14 | <0,001 | 0,099 |
|  |  | 21 | 3,88E-07 | 0,004 |
| JQ1 | 50 | 3 | 0,913 | 0,154 |
|  |  | 7 | 0,005 | 0,177 |
|  |  | 14 | 0,083 | 0,010 |
|  |  | 21 | 0,936 | 0,059 |
|  | 100 | 3 | 0,569 | 0,081 |
|  |  | 7 | 0,343 | 0,001 |
|  |  | 14 | 0,002 | 0,003 |
|  |  | 21 | 0,153 | 0,018 |
|  | 200 | 3 | 0,143 | 0,019 |
|  |  | 7 | 0,193 | 0,002 |
|  |  | 14 | 0,005 | 0,114 |
|  |  | 21 | 0,004 | 0,008 |

**
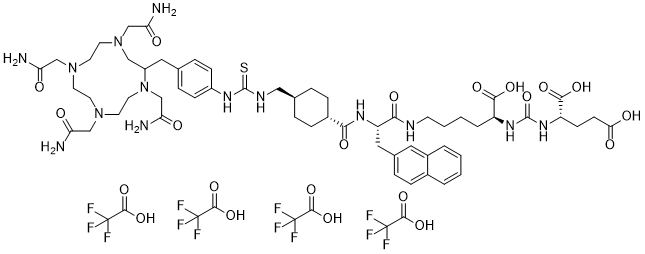
**

**Supplementary Figure 1.** Chemical structures of AB001 (p-SCN-Bn-TCMC-PSMA) trifluoracetic acid salt.


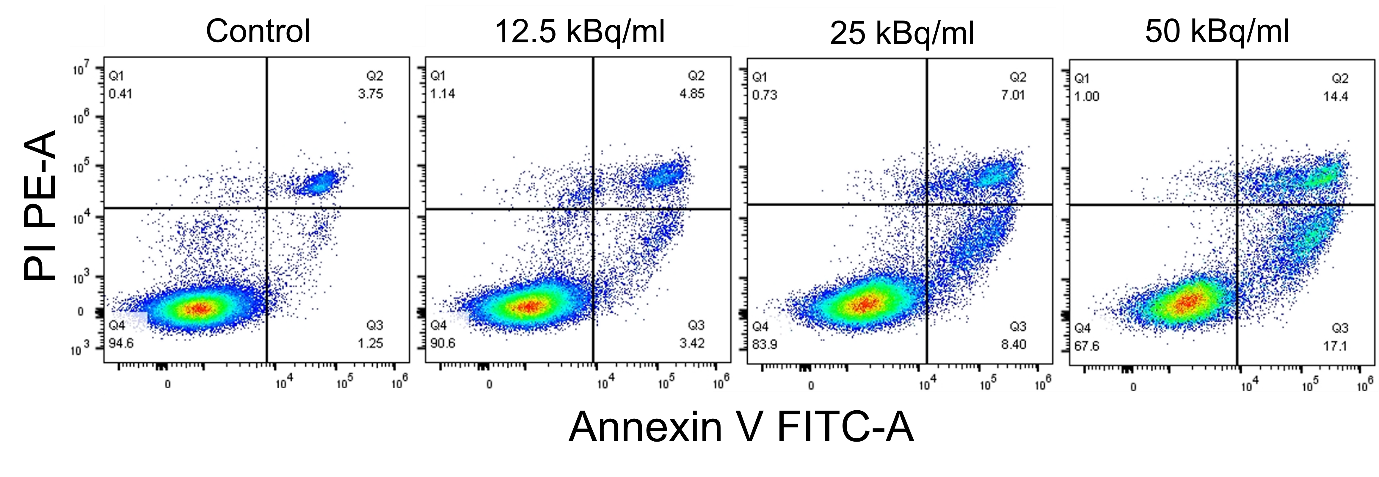


**Supplementary Figure 2.** Representative gating strategy for viability analysis with flow cytometry. Viability was assessed by using Annexin V FITC and propidium iodide (PI) staining on days 1, 3 and 6 to distinguish viable, apoptotic and necrotic cells. Illustrated in the figure are groups treated with [^212^Pb]Pb-AB001 (4-hour incubation) 6 days post treatment.


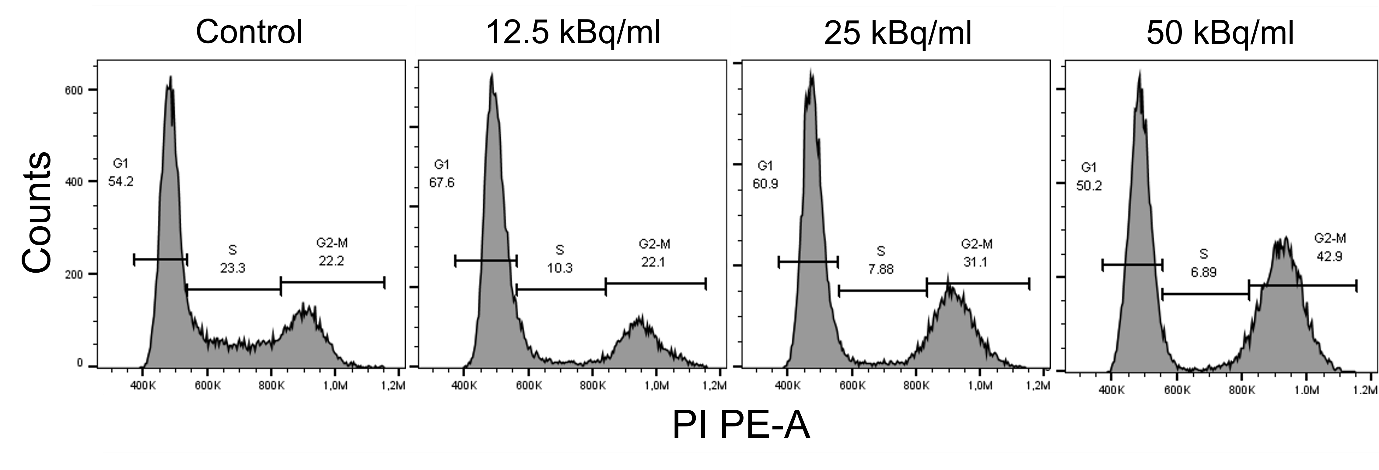


**Supplementary Figure 3.** Representative gating strategy for cell cycle analysis with flow cytometry. Illustrated in the figure are groups treated with [^212^Pb]Pb-AB001 (4-hour incubation) 1 day post treatment.
